# Dynamics of community-acquired meningitis syndrome outbreaks in southern France

**DOI:** 10.3389/fmicb.2022.1102130

**Published:** 2023-01-26

**Authors:** Madjid Morsli, Florian Salipante, Quentin Kerharo, Agathe Boudet, Robin Stephan, Catherine Dunyach-Remy, Christine Zandotti, Jean-Philippe Lavigne, Michel Drancourt

**Affiliations:** ^1^IHU Méditerranée Infection, Marseille, France; ^2^Aix-Marseille-Université, IRD, MEPHI, IHU Méditerranée Infection, Marseille, France; ^3^Laboratoire de biostatistique, Épidémiologie Clinique, Santé Publique, Innovation et Méthodologie, CHU de Nîmes, Université de Montpellier, Nîmes, France; ^4^Laboratoire de Microbiologie, Assistance Publique-Hôpitaux de Marseille, IHU Méditerranée Infection, Marseille, France; ^5^VBIC, INSERM U1047, Université de Montpellier, Service de Microbiologie et Hygiène Hospitalière, CHU Nîmes, Nîmes, France

**Keywords:** community-acquired meningitis, cerebrospinal fluid, etiology, outbreak, nondocumented meningitis, season, dynamics

## Abstract

In southern France, cases of community-acquired meningitis syndrome (CAM) are typically clustered as outbreaks with determinants which remain unknown. This 61-month retrospective investigation in Nîmes and Marseille university hospital laboratories, yielded 2,209/20,779 (10.63%) documented CAM cases caused by 62 different micro-organisms, represented by seasonal viral etiologies (78.8%), including Enterovirus, *Herpes Simplex Virus* (HSV), and *Varicella-Zoster Virus* (VZV; 1,620/2,209 = 73.4%). Multi correspondence analysis revealed an association of infection with age and sex, with the risk of infection being relatively higher in young men, as confirmed by Fisher’s exact test (*p* < 10^−3^). Bacterial meningitis accounted for 20% of cases, mostly caused by *Streptococcus pneumoniae* (27.4% of cases), *Neisseria meningitidis* (12.5%), and *Haemophilus influenzae* (9.5%) with bacteria/virus coinfection (0.9%), and only six cases of documented fungal meningitis. In total, 62.6% of cases, of which 88.7% were undocumented, arose from 10 outbreaks. 33.2% of undocumented cases were aged >60 years compared to 19.2% of documented cases (*p* < 0.001), and viral infection was more common in the summer (87.5%) compared to other seasons (72.3%; *p* < 0.001). Outbreaks most often started in Nîmes and moved eastward toward Marseille at a speed of ~9 km/day, and these dynamics significantly correlated with atmospheric temperature, especially during summer outbreaks. In particular, the incidence of Enterovirus-driven outbreaks correlated with temperature, revealing correlation coefficients of 0.64 in Nîmes and 0.72 in Marseille, and its occurrence in Marseille lagged that in Nîmes by 1–2 weeks. Tracing the dynamics of CAM outbreak during this retrospective investigation in southern France yielded a speed of displacement that correlated with the variation in temperature between both cities, and these results provide clues for the next occurrence of undocumented outbreaks.

## Introduction

Community-acquired infectious meningitis (CAM) affects more than 1.3 million patients every year worldwide, and has a 40% lethality rate, partially depending on the causative pathogen (Bijlsma et al., 2016; Sulaiman et al., 2017; Broberg et al., 2018). RNA viruses, chiefly Enterovirus, are the most frequently documented pathogens and are most responsible for benign meningitis, but rarely cause disability and life-threatening cases following progression of the infection to encephalitis (Broberg et al., 2018). DNA viruses are mainly responsible for disability and life-threatening infections (Guan et al., 2015; Bradshaw and Venkatesan, 2016). Bacterial meningitis is a deadly form of meningitis, causing more than 50% of annual deaths from all-cause meningitis (290,000) and leaving one in five people who recover with a chronic neurological disorder (Rodgers et al., 2020). However, 40–60% of CNS-infecting cases had an unknown etiology (Sigfrid et al., 2019). While CAM may evolve as sporadic cases, CAM outbreaks have been well described for specific pathogens over a well-defined period of time (Polage and Cohen, 2016; van de Beek et al., 2016; Shukla et al., 2017; Broberg et al., 2018; Matulyte et al., 2020). In Europe, Enterovirus outbreaks are recorded annually in the summer (Harvala et al., 2017; Broberg et al., 2018), causing 4,537 meningitis cases in 15 European countries in 2018 and primarily infecting young people (Broberg et al., 2018), while the incidence of herpes encephalitis is estimated at 2–4 cases/1,000,000 people worldwide, mainly infecting older people aged >50 years (Bradshaw and Venkatesan, 2016). Bacterial outbreaks are usually associated with *Haemophilus influenzae* (*H. influenzae*), *Streptococcus pneumoniae* (*S. pneumoniae*), and *Neisseria meningitidis* (*N. meningitidis*) infections, causing 16 million cases between 1990 and 2013, with a high incidence in sub-Saharan Africa (Global Burden, 2015; Van de Beek et al., 2016). Sporadic cases of infection with other bacteria with low infectious prevalence can be associated with CAM, depending on the region and population (Van de Beek et al., 2016).

Here, investigating a large retrospective series of data from CAM syndrome patients diagnosed in two university hospitals in southern France provided a unique opportunity to describe the temporal and spatial dynamics of outbreaks of CAM syndrome in this region, yielding a previously unreported finding regarding the climate-driven eastward movement of CAM outbreaks in which documented cases masked undocumented ones. These results provide some analogical clues to the next occurrence of undocumented outbreaks.

## Patients and methods

### Ethical statement

This retrospective study (December 2014 to December 2019) only collected anonymous data issued from routinely diagnosed cases originating from the University Hospital Institute (IHU) Méditerranée Infection Laboratory, Marseille, and the Department of Microbiology and Hospital Hygiene, Nîmes, located approximately 120 km apart in southern France. This study was retrospective and anonymous and required no specific intervention for any patient and no specific clinical samples. Accordingly, this study was approved by the Ethics Committee of the Institut Méditerranée Infection under numbers 2021-004 (Ethics Committee of the IHU Méditerranée Infection, Marseille) and 21.03.11 (Interface Recherche Bioéthique Institutional Review Board Ethics Committee, CHU, Nîmes).

### Data selection and setting

For each year, approximately 3,500 cerebrospinal fluid (CSF) samples from the Assistance Publique-Hôpitaux de Marseille (APHM) were received at the point-of-care (POC) laboratory of the IHU Méditerranée Infection in Marseille, and between 900 and 1,500 CSF samples were investigated at the Department of Microbiology and Hospital Hygiene at Nîmes University Hospital. Only CSF samples from patients clinically suspected of having community-acquired meningitis (infections contracted outside the hospital and diagnosed within 48 h of admission) were included in this study, and all CSF samples from patients undergoing neurosurgery, transplantation, or dialysis, and who were hospitalized for more than 48 h were excluded from the final database. Additionally, only data from patients with complete clinical records, including age, sex, sampling date, and final diagnosis, were analyzed (Appendix 1).

### Routine laboratory diagnosis

Cerebrospinal fluid (CSF) specimens routinely submitted to the POC laboratory of the two hospitals were examined to measure leukocytes and red blood cells using the NucleoCounter® NC-3000™ apparatus and NucleoView™ software (ChemoMetec Inc., Allerod, Denmark). At the same time, CSF was incorporated into the FilmArray® ME Panel assay (bioMérieux, Marcy-l’Etoile, France) for the multiplex PCR-based detection of 14 pathogens, as previously described (Boudet et al., 2019; Vincent et al., 2020). Then, depending on the POC primarily diagnostic test, real-time PCR (RT–PCR) and culture were routinely performed to confirm and complete the POC diagnosis, as well as to remove false-positives generated by the commercial PCRs (Naccache et al., 2018; Boudet et al., 2019). For any further molecular diagnosis, nucleic acids were extracted from 200 μl of CSF using the EZ1 DNA Kit and the EZ1 Virus Mini Kit v2.0 (Qiagen, Courtaboeuf, France), and any remaining extracted DNA was stored at −80°C. Target amplification using 43 cycles of RT–PCR was performed in the LightCycler® 480 thermal cycler (Roche, Meylan, France) using a specific program for each targeted pathogen and incorporating 5 μl of RNA and LC480 Probes MasterMix 2X (Roche) or 5 μl of DNA and Takyon No Roxe Probe MasterMix (Eurogentec, Angers, France) in a 20-μl final reaction volume. CSF culture was systematically performed using both Chocolate agar PolyViteX (bioMérieux) and Columbia agar enriched with 5% sheep blood (bioMérieux) media incubated at 37°C under 5% CO_2_ for 5 days (Société Française de Microbiologie, 2018). In addition, Columbia agar enriched with 5% sheep blood (bioMérieux) was inoculated with CSF and incubated in anaerobic conditions for 10 days at 37°C to select for anaerobic pathogens. Cultured microorganisms were identified by using matrix-assisted laser desorption/ionization time-of-flight mass spectrometry as previously described (Seng et al., 2009), spectra were compared against an enriched Biotyper identification database (Bruker Daltonics, Bremen, Germany) in Marseille and against a VITEK® MS (version 3.2) database (bioMérieux) in Nimes. In addition to a routine pathogen panel, arthropod-borne viruses, including West-Nile-Virus, Toscana Virus, and Usutu Virus, were systematically investigated every year between May 1 and November 30, according to the French regional health agency surveillance program, and outside this period according to the recommendations of clinicians.^1^

### Outbreak definition

The time series of incidence was first smoothed with a moving average using a 9-week window. Then, to make the data across cities comparable, the smoothed time series were standardized (centering and scaling). An outbreak period was defined when the maximum standardized value of incidence was equal to or greater than 0.5 in each period in at least one of the two cities’ series. The outbreak start-point was defined as the date when the standardized value of incidence rose above baseline (0), and the outbreak stop-point was defined as the date when the standardized value of incidence fell below baseline. For cases of complex outbreaks featuring different behaviors (e.g., outbreaks 6 and 8), the period was set manually. Finally, climatic data recovered from the French national weather registry,^2^ including temperature, humidity, and wind, were superimposed over the epidemiological data to test for any significant correlation with outbreak patterns.

### Statistical analyses

R software version (3.6.1; R Development Core Team, 2019) was used for all the statistical analyses in this work. All statistical tests were two sided, and the type one error rate was set to 0.05. The quantitative variables are reported as the mean ± SD, and qualitative variables are reported as *N* (%). For two-group comparisons (e.g., across the two hospitals), Mann–Whitney or Student’s *t*-tests were used (as appropriate) for quantitative variables, and Chi-squared or Fisher’s exact tests were used (as appropriate) for qualitative variables. The test results were first considered as binary (positive or documented/negative or undocumented) and were evaluated according to location (Nîmes, Marseille), season of outbreak, and the patients’ sex and age. To compare the positivity status using a multivariate analysis (age, sex, and season city), a logistical regression model was used, and the adjusted odds ratio (AOR) was reported. The test results were then considered within the context of the identified pathogens. Multiple correspondence analysis (MCA) was performed with the FactoMineR package (Jombart, 2008) to explore the relationships between the incidence of specific pathogens with the patients’ age group and sex and the location, season, and period of the outbreak. To track the outbreak patterns of documented cases, cases of documented etiologies were placed into three major groups: DNA viruses, RNA viruses, and bacteria. The observations obtained with MCA were confirmed by Fisher’s exact test when necessary. In a subsequent analysis, to track the outbreak patterns of documented cases, cases of documented etiologies were placed into three major groups: DNA viruses, RNA viruses, and bacteria. To better explore the trends and seasonality of the series, data were then managed as time series using the stats and forecast packages in R (Hyndman and Khandakar, 2008) in particular to define the outbreak periods (as previously mentioned) and to compute the average time period between infections occurring in Nîmes and Marseille regarding the occurrence of outbreaks and temperatures.

## Results

### General data

**Figure 1:**
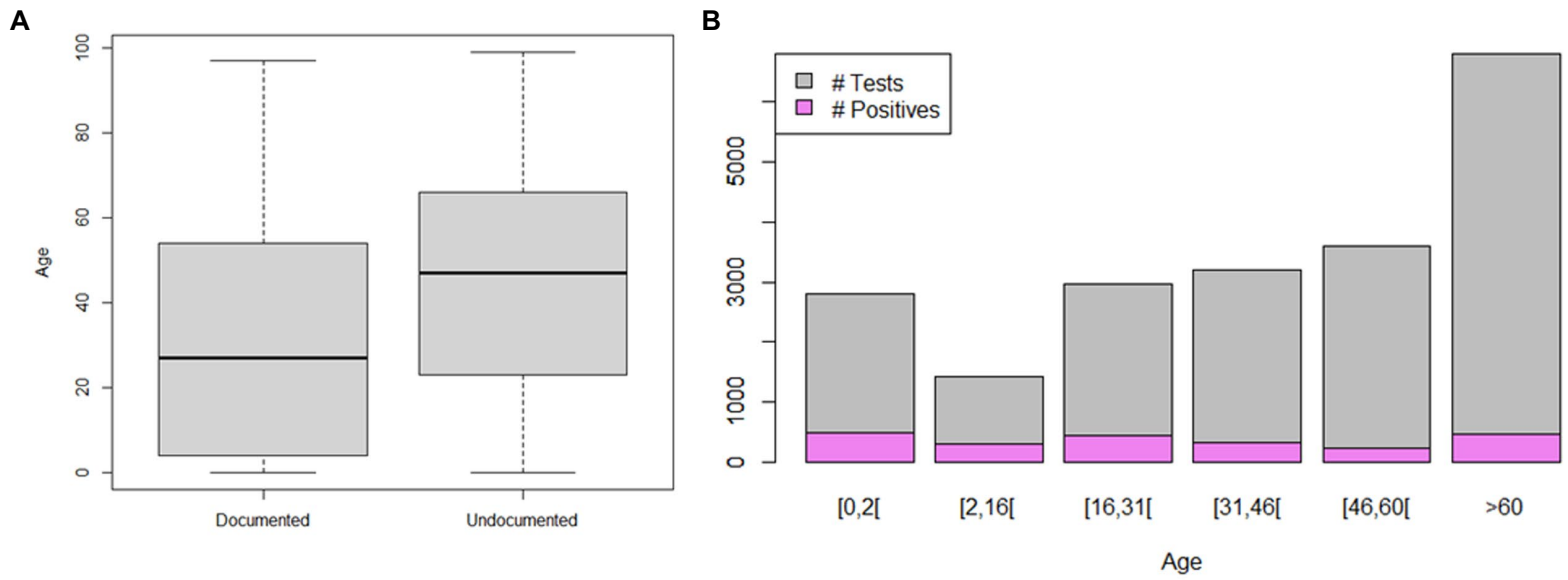
Distribution of meningitis cases according to patient age. **(A)** Boxplot of total negative (undocumented) and positive (documented) meningitis cases according to patient age. **(B)** Bar plot of meningitis cases and positive diagnoses according to patient age groups.

Based on the inclusion criteria, 20,779/28,495 (72.9%) of CSF samples (one CSF sample per patient presenting with a meningitis syndrome) investigated between December 2014 and December 2019 at the IHU Méditerranée Infection, Marseille (15,246 CSFs) and CHU Nîmes (5,533 CSFs) laboratories were retrospectively included over this 61-month study held between December 1, 2014 and December 31, 2019. There was a general upward trend in the number of documented cases since 2017 in both cities, although we observed a difference in the behavior of the two cities regarding the number of undocumented cases, which strongly decreased in 2016 and returned to baseline in 2017 in Nîmes but increased from mid-2018 onwards in Marseille. Overall, males formed a marginal majority (52.77%; 54.3% in Marseille, 48.6% in Nîmes; *p* < 10^−4^), while females were the majority specifically among documented cases (49.4% male patients; 54.5% in Marseille and 42.8% in Nîmes; *p* < 10^−4^; Table 1). Additionally, the CAM population was younger in Marseille than in Nîmes (mean age: 40.7 ± 27.2 years vs. 47.2 ± 25.6, respectively; *p* < 0.001; Table 1), but this difference did not have any detectably significant influence on the fact that the overall prevalence of documented CAM cases significantly decreased with age for each age group when compared to the prevalence of the reference age group of (0–2) years, except in the patients in the 2–16 years age group, which presented a higher prevalence (adjusted odds ratio, AOR = 0.85, *p* = 0.0461; Figure 1). Logistical regression results showed that the number of documented cases was significantly higher in Nîmes that in Marseille (AOR = 2.8, *p* < 0.001), in females than in males (AOR = 1.13, *p* = 0.01), and in the 0–2 and 2–16 years age groups than in the other age groups (AOR = 1.38, 2.18, 3.69, *p* < 0.001). The most exhaustively diagnosed age group was that aged 2–16 years (AOR = 0.85, *p* = 0.11; Figure 1). This observation was confirmed by the Chi-squared test, highlighting a significant difference in the distribution of ages between total (documented plus nondocumented) CAM cases and documented CAM cases (*p* < 0.001).

**Table 1:** Summary statistics for the age and gender variables, comparisons according to city and positive/negative status.

		Global tested population				Positive result population				
		Total	Marseille	Nimes	*p*-value (Nimes vs Marseille)	Total	Marseille	Nimes	*p*-value (Nimes vs Marseille)	*p*-value (Global vs Positives)
N		20,779 (100%)	15,246 (73.4%)	5,533 (26.6%)	-	2,209	1,238	971	-	<0.001
Gender (Men)		10,966 (52.77%)	8,274 (54.27%)	2,692 (48.65%)	<0.001	1,091 (49.39%)	675 (54.52%)	416 (42.84%)	<0.001	<0.001
Age		42.46 ± 26.94	40.75 ± 27.22	47.19 ± 25.57	<0.001	31.43 ± 27.56	27.4 ± 2 6.84	36.57 ± 27.62	<0.001	<0.001
Bacteria		-	-	-	-	471 (21.32%)	376 (30.37%)	95 (9.78%)	<0.001	-
DNA Viruses		-	-	-	-	934 (42.28%)	296 (23.91%)	638 (65.70%)		
Fungi		-	-	-	-	6 (0.27%)	6 (0.48%)	0 (0%)		
RNA Viruses		-	-	-	-	847 (38.34%)	599 (48.38%)	248 (25.54%)		

### General epidemiology

**Figure 2:**
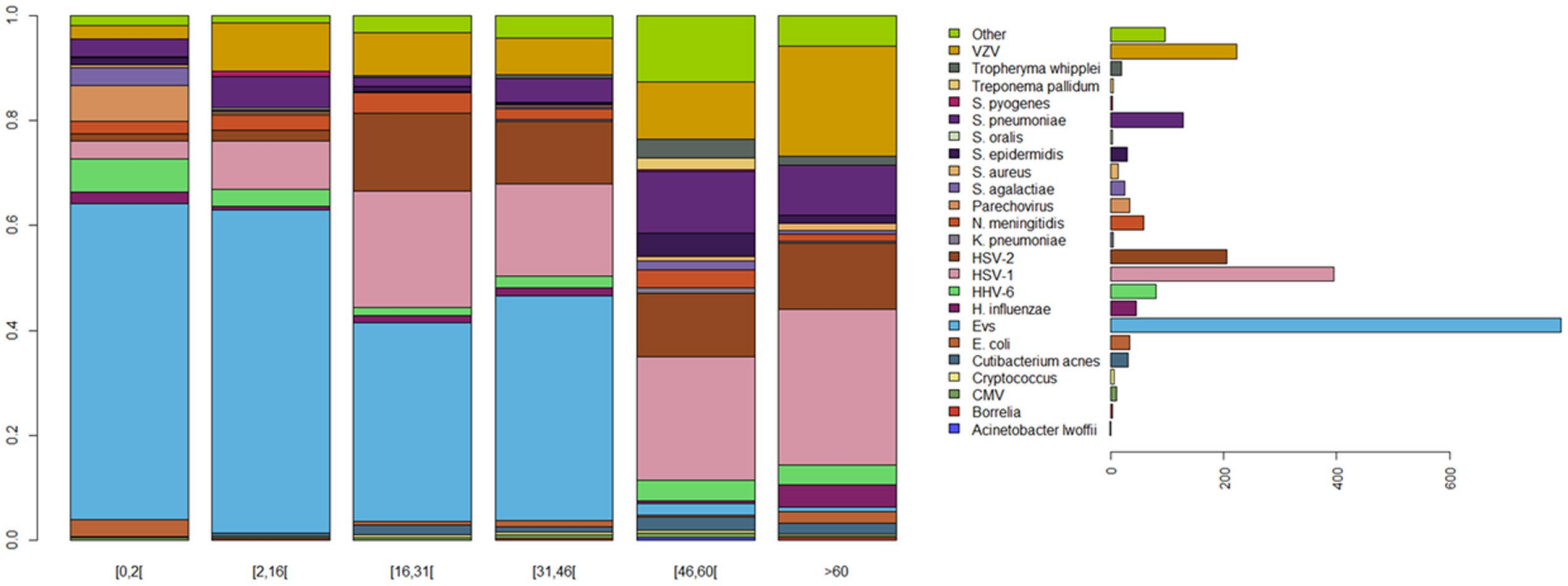
Comparison of the microbial diversity of community-acquired meningitis. Patient age-dependent pathogen diversity: patients aged 0–2 and 2–16 were mostly diagnosed as positive for Enterovirus, the majority of patients aged 16–46 years old were positive for Enterovirus, HSV-1 and HSV-2, patients >46 years were positive for HSV-1, HSV-2, VZV, and bacterial infections. The legend shows the total abundance of each pathogen. Pathogens with global abundance less than 1% are gathered in group “Other.” Evs, Enterovirus; HSV-1, Herpes Simplex Virus 1; HSV-2, Herpes Simplex Virus 2; VZV, Varicella Zoster Virus; and HHV-6, Human Herpes Virus 6.

A total of 2,209 (10.63%) patients were documented in both centers after laboratory investigations, as reported above. 1,762 (78.8%) patients had viral meningitis, including 17 coinfections with virus/virus (0.8%), 18 coinfections with virus/bacteria (0.8%), one coinfection with virus/bacteria/bacteria (0.04%), and two coinfections with virus/virus/bacteria (0.09%). 461 patients had bacterial meningitis (20.9%), including nine coinfections with bacteria/bacteria (0.4%), one coinfection with virus/bacteria/bacteria (0.04%), and 18 coinfections with virus/bacteria (0.8%). Six patients had fungal meningitis (0.3%; Table 2; Appendix 1). CAM was caused by a total of 62 different microorganisms, including 49 bacteria (79%), 12 viruses (19%, seven DNA and five RNA viruses), and one fungal pathogen (*Cryptococcus* 2%), each with variable contributions to CAMs (Figure 2; Table 2). Viruses were the main causative pathogens, and 12 viral species were identified in 79.8% of documented CSFs, mainly Enteroviruses (797, 36.1%), HSV-1 (395, 17.9%), HSV-2 (205, 9.3%), and VZV (223, 10.1%; Figure 2; Table 2). Dengue and BK viruses, JC virus, Toscana virus, and West Nile virus were detected at a very low frequency in the studied population (Table 2). *Streptococcus pneumoniae* (129, 5.8%), *N. meningitidis* (59, 2.7%), and *H. influenzae* (45, 2%) were the most prevalent bacteria, followed by *Escherichia coli* (34, 1.5%), *Cutibacterium acnes* (31, 1.4%), *Staphylococcus epidermidis* (30, 1.4%), and *Streptococcus agalactiae* (*S. agalactiae*; 25, 1.1%). *Tropheryma whipplei*, *Staphylococcus aureus*, and *Listeria monocytogenes* were detected in rare cases. Additionally, only six cases of *Cryptococcus* meningitis were detected, all in immunocompromised patients (Table 2).

**Table 2:** Prevalence of pathogens by age for the period spanning from December 2014 to December 2019 (Data gathered from Nîmes and Marseille).

	**[0,2]**	**[2,16]**	**[16,31]**	**[31,46]**	**[46,60]**	**>60**	**Total**
Enterovirus	291 (36.5%)	186 (23.3%)	170 (21.3%)	141 (17.7%)	5 (0.6%)	4 (0.5%)	797
Herpes Simplex Virus 1	17 (4.3%)	28 (7.1%)	99 (25.1%)	58 (14.7%)	54 (13.7%)	139 (35.2%)	395
Varicella Zoster Virus	12 (5.4%)	28 (12.6%)	37 (16.6%)	23 (10.3%)	25 (11.2%)	98 (43.9%)	223
Herpes Simplex Virus 2	6 (2.9%)	6 (2.9%)	67 (32.7%)	39 (19%)	28 (13.7%)	59 (28.8%)	205
*Streptococcus pneumoniae*	17 (13.2%)	18 (14%)	7 (5.4%)	15 (11.6%)	27 (20.9%)	45 (34.9%)	129
Human Herpesvirus Virus 6	30 (37%)	10 (12.3%)	7 (8.6%)	7 (8.6%)	9 (11.1%)	18 (22.2%)	81
*Neisseria meningitidis*	12 (20.3%)	9 (15.3%)	17 (28.8%)	7 (11.9%)	8 (13.6%)	6 (10.2%)	59
*Haemophilus influenzae*	11 (24.4%)	2 (4.4%)	6 (13.3%)	5 (11.1%)	1 (2.2%)	20 (44.4%)	45
*Escherichia coli*	16 (47.1%)	0 (0%)	3 (8.8%)	4 (11.8%)	1 (2.9%)	10 (29.4%)	34
Human parechovirus	33 (97.1%)	1 (2.9%)	0 (0%)	0 (0%)	0 (0%)	0 (0%)	34
*Cutibacterium acnes*	1 (3.2%)	2 (6.5%)	9 (29%)	3 (9.7%)	6 (19.4%)	10 (32.3%)	31
*Staphylococcus epidermidis*	6 (20%)	1 (3.3%)	5 (16.7%)	1 (3.3%)	10 (33.3%)	7 (23.3%)	30
*Streptococcus agalactiae*	16 (64%)	0 (0%)	1 (4%)	1 (4%)	4 (16%)	3 (12%)	25
*Tropheryma whipplei*	0 (0%)	0 (0%)	2 (10%)	2 (10%)	8 (40%)	8 (40%)	20
*Staphylococcus aureus*	3 (21.4%)	1 (7.1%)	0 (0%)	1 (7.1%)	2 (14.3%)	7 (50%)	14
JC Virus	0 (0%)	0 (0%)	0 (0%)	0 (0%)	4 (33.3%)	8 (66.7%)	12
Cytomegalovirus	2 (18.2%)	1 (9.1%)	2 (18.2%)	2 (18.2%)	2 (18.2%)	2 (18.2%)	11
Dengue virus	0 (0%)	1 (10%)	4 (40%)	2 (20%)	3 (30%)	0 (0%)	10
*Staphylococcus hominis*	2 (22.2%)	0 (0%)	1 (11.1%)	2 (22.2%)	1 (11.1%)	3 (33.3%)	9
BK Virus	0 (0%)	1 (14.3%)	0 (0%)	1 (14.3%)	2 (28.6%)	3 (42.9%)	7
*Listeria monocytogenes*	0 (0%)	0 (0%)	0 (0%)	1 (14.3%)	2 (28.6%)	4 (57.1%)	7
*Cryptococcus meoformans/gattii*	0 (0%)	0 (0%)	2 (33.3%)	2 (33.3%)	1 (16.7%)	1 (16.7%)	6
*Klebsiella pneumoniae*	0 (0%)	0 (0%)	0 (0%)	1 (20%)	2 (40%)	2 (40%)	5
*Staphylococcus capitis*	2 (40%)	0 (0%)	1 (20%)	0 (0%)	2 (40%)	0 (0%)	5
Toscana virus	0 (0%)	0 (0%)	2 (40%)	0 (0%)	2 (40%)	1 (20%)	5
*Treponema pallidum*	0 (0%)	0 (0%)	0 (0%)	0 (0%)	5 (100%)	0 (0%)	5
*Borrelia* sp.	0 (0%)	1 (25%)	0 (0%)	1 (25%)	0 (0%)	2 (50%)	4
*Streptococcus pyogenes*	0 (0%)	3 (75%)	0 (0%)	0 (0%)	1 (25%)	0 (0%)	4
*Enterococcus faecalis*	0 (0%)	1 (33.3%)	0 (0%)	0 (0%)	2 (66.7%)	0 (0%)	3
*Pseudomonas aeruginosa*	1 (33.3%)	1 (33.3%)	0 (0%)	0 (0%)	0 (0%)	1 (33.3%)	3
*Staphylococcus haemolyticus*	0 (0%)	0 (0%)	0 (0%)	1 (33.3%)	1 (33.3%)	1 (33.3%)	3
*Streptococcus oralis*	1 (33.3%)	1 (33.3%)	0 (0%)	1 (33.3%)	0 (0%)	0 (0%)	3
*Acinetobacter baumannii*	0 (0%)	0 (0%)	1 (50%)	1 (50%)	0 (0%)	0 (0%)	2
*Enterobacter cloacae*	0 (0%)	0 (0%)	1 (50%)	0 (0%)	1 (50%)	0 (0%)	2
*Propionibacterium avidum*	0 (0%)	0 (0%)	0 (0%)	1 (50%)	0 (0%)	1 (50%)	2
*Ureaplasma urealyticum*	2 (100%)	0 (0%)	0 (0%)	0 (0%)	0 (0%)	0 (0%)	2
*Acinetobacter lwoffii*	0 (0%)	0 (0%)	0 (0%)	0 (0%)	1 (100%)	0 (0%)	1
*Acinetobacter radioresistens*	0 (0%)	0 (0%)	0 (0%)	0 (0%)	0 (0%)	1 (100%)	1
*Bacillus fragilis*	0 (0%)	0 (0%)	0 (0%)	0 (0%)	0 (0%)	1 (100%)	1
*Bacillus megaterium*	0 (0%)	0 (0%)	0 (0%)	1 (100%)	0 (0%)	0 (0%)	1
*Bacillus simplex*	0 (0%)	0 (0%)	0 (0%)	0 (0%)	1 (100%)	0 (0%)	1
*Chlamydophila pneumoniae*	0 (0%)	0 (0%)	1 (100%)	0 (0%)	0 (0%)	0 (0%)	1
*Citrobacter freundii*	0 (0%)	0 (0%)	0 (0%)	0 (0%)	0 (0%)	1 (100%)	1
*Citrobacter koseri*	1 (100%)	0 (0%)	0 (0%)	0 (0%)	0 (0%)	0 (0%)	1
*Kocuria rhizophila*	0 (0%)	0 (0%)	1 (100%)	0 (0%)	0 (0%)	0 (0%)	1
*Mycobacterium tuberculosis*	0 (0%)	0 (0%)	0 (0%)	0 (0%)	1 (100%)	0 (0%)	1
*Pantoea* sp.	0 (0%)	0 (0%)	0 (0%)	0 (0%)	1 (100%)	0 (0%)	1
*Parvimonas micra*	0 (0%)	0 (0%)	0 (0%)	0 (0%)	1 (100%)	0 (0%)	1
*Pasteurella multocida*	0 (0%)	0 (0%)	1 (100%)	0 (0%)	0 (0%)	0 (0%)	1
*Proteus mirabilis*	0 (0%)	0 (0%)	0 (0%)	1 (100%)	0 (0%)	0 (0%)	1
*Proteus vulgaris*	0 (0%)	0 (0%)	0 (0%)	0 (0%)	1 (100%)	0 (0%)	1
Roseomonas sp	0 (0%)	0 (0%)	0 (0%)	0 (0%)	1 (100%)	0 (0%)	1
*Streptococcus anginosus*	0 (0%)	0 (0%)	0 (0%)	0 (0%)	1 (100%)	0 (0%)	1
*Staphylococcus intermedius*	0 (0%)	0 (0%)	0 (0%)	0 (0%)	0 (0%)	1 (100%)	1
*Staphylococcus lugdunensis*	0 (0%)	0 (0%)	0 (0%)	0 (0%)	1 (100%)	0 (0%)	1
*Serratia marcescens*	0 (0%)	0 (0%)	0 (0%)	0 (0%)	1 (100%)	0 (0%)	1
*Streptococcus mitis*	1 (100%)	0 (0%)	0 (0%)	0 (0%)	0 (0%)	0 (0%)	1
*Streptococcus parasanguinis*	0 (0%)	0 (0%)	1 (100%)	0 (0%)	0 (0%)	0 (0%)	1
*Staphylococcus pasteuri*	0 (0%)	0 (0%)	0 (0%)	1 (100%)	0 (0%)	0 (0%)	1
*Streptococcus salivarius*	0 (0%)	0 (0%)	0 (0%)	0 (0%)	0 (0%)	1 (100%)	1
*Staphylococcus warneri*	0 (0%)	0 (0%)	0 (0%)	1 (100%)	0 (0%)	0 (0%)	1
Virus West Nile	0 (0%)	0 (0%)	0 (0%)	1 (100%)	0 (0%)	0 (0%)	1
Total	483 (21.4%)	302 (13.4%)	448 (19.8%)	328 (14.5%)	229 (10.1%)	468 (20.7%)	2,258 (100%)

**Figure 3:**
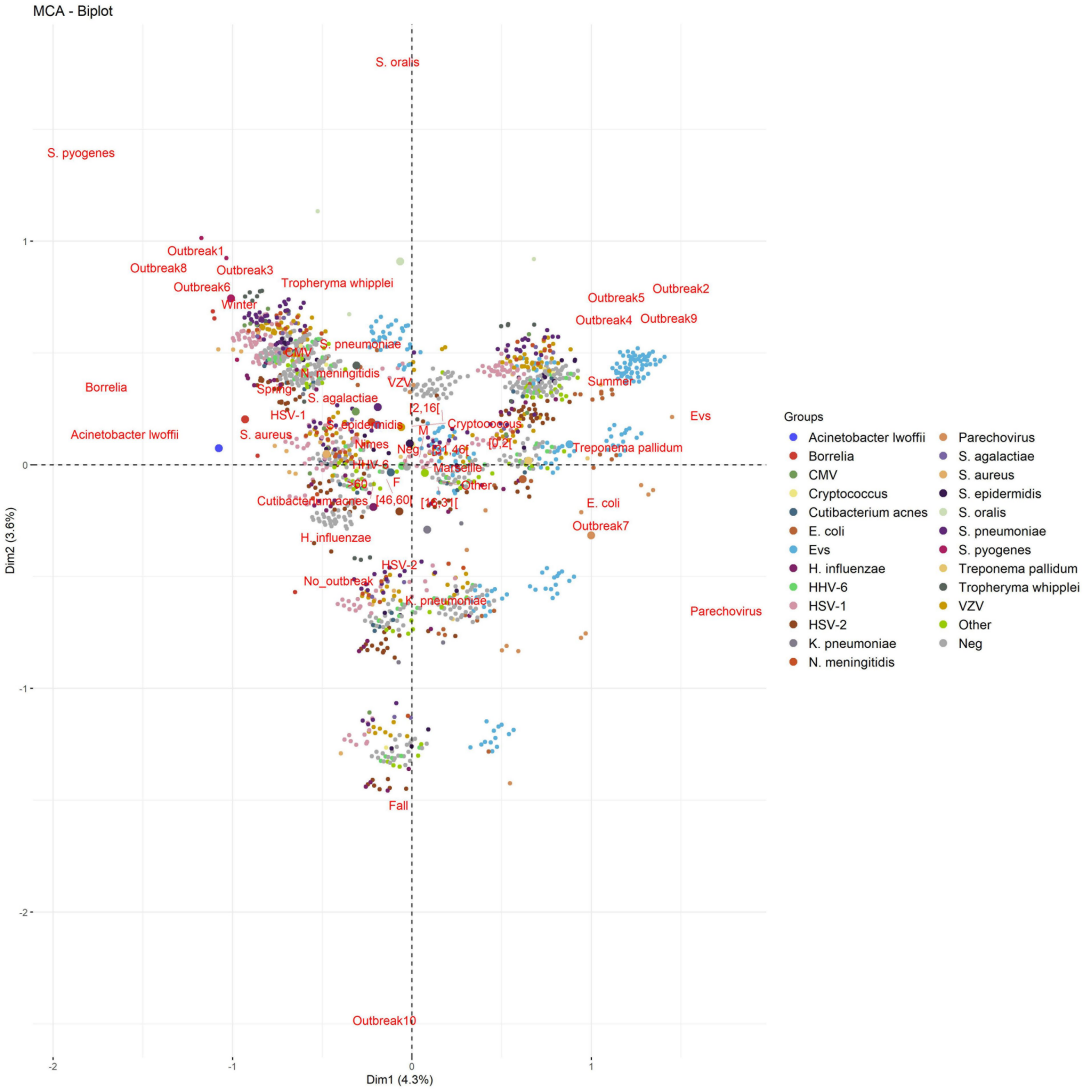
Multiple correspondence analysis of positive/negative (documented/undocumented) cases, pathogens, and season according to the different outbreaks. Infections in Marseille were more closely associated with summer outbreaks and younger patients, whereas infections in Nîmes were more associated with winter outbreaks and older patients, mostly HSV-1, HSV-2, VZV, and *Haemophilus influenzae*. There was no notable difference concerning sex.

Relationships were noted between age and pathogen type. Enterovirus was prevalent in patients aged <16 years (59.8%); HHV-6 in patients aged 0–16 and > 60 years (71.6%); HSV1, HSV-2, and VZV in patients aged 16–31 and > 60 years (60.2, 61.5, and 60.5%, respectively); *H. influenzae* in patients aged 0–2 and > 60 years (68.9%); *S. pneumoniae* in patients aged >45 years (67.4%); *N. meningitidis* in patients aged <30 years (64.4%); and Human parechovirus and *S. agalactiae* in patients aged 0–2 years (97 and 64%, respectively; Table 2; Figures 2, 3). Moreover, MCA indicated a preferential association between sex and the causative pathogen, as HSV-1 and HSV-2 were mainly documented in older female patients (73.7 and 57.7% and *p* < 10^−13^ and *p* < 10^−4^, respectively), whereas Enterovirus infection was mostly identified among young male patients (55.7%, *p* = 0.12).

Finally, some seasonal infections, as illustrated by MCA investigation and confirmed by statistical analyses for significance, were observed during the study period for some pathogens (*p* < 0.001), including Enterovirus and Human parechovirus, which were mainly documented in summer and autumn, and *N. meningitidis*, *S. pneumoniae*, *S. agalactiae*, HSV-1, HSV-2, VZV, and Cytomegalovirus, which were mainly documented in winter and spring (Figure 3, Appendix 3).

### Outbreaks

**Figure 4:**
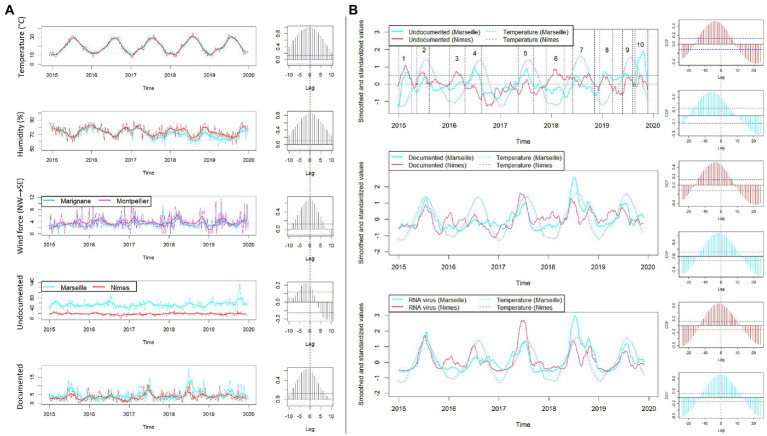
Synchronization of outbreaks and weather data between Marseille and Nîmes by time series analysis. **(A)** The time series for temperatures, humidity, and wind force (in Marignane and Montpellier, two cities close to Marseille and Nimes), undocumented and documented cases are represented for both Marseille (in blue) and Nimes (in red). Each time series is represented in its real values (thin lines) and smoothed with moving average using a window size of 9 weeks (thick lines). To the right of each time series graphic, the associated cross-correlation function plot comparing Nîmes and Marseille at different lags is shown. A high correlation at a negative lag should be interpreted as an event occurring first in Nîmes and then in Marseille. **(B)** Time series were first smoothed using moving average with a window size of 9 weeks. Then, in order to make the cities and different variables more comparable, a standardization was applied (cantering and scaling). The 10 periods of the outbreaks were defined based on undocumented cases (graphic on the top). The standardized and smoothed time series of undocumented cases, documented cases and specifically RNA virus cases are shown in the three graphics (thick red and blue lines) along with standardized and smoothed temperatures in Nîmes and Marseille (red and blue thin dotted lines). To the right each time series graphic, the associated cross-correlation function plot comparing temperature and undocumented, documented and RNA virus, respectively (from top to bottom), are shown in red for Nîmes and in blue for Marseille.

Ten outbreaks as defined by the criteria above, comprising 12,919/20,779 (62.2%) patients and 1,438/2,209 (65.1%) documented cases, were observed over 61 months (Figure 4A). There was a trend toward an increasing frequency of outbreak occurrence over these 61 months. Notably, outbreaks mainly started in Nîmes and emerged eastward in Marseille with an average lag of 1–2 weeks (~13 days). This observation was confirmed with the use of time series decomposition (using the decompose function of the stats package in R) and the use of a cross-correlation function performed on the seasonal regions of the decompositions of the Nîmes and Marseille series. Given that the two cities are 120 km apart, we measured an average eastward displacement speed of ~0.9 km/day (Figure 4A).

**Figure 5:**
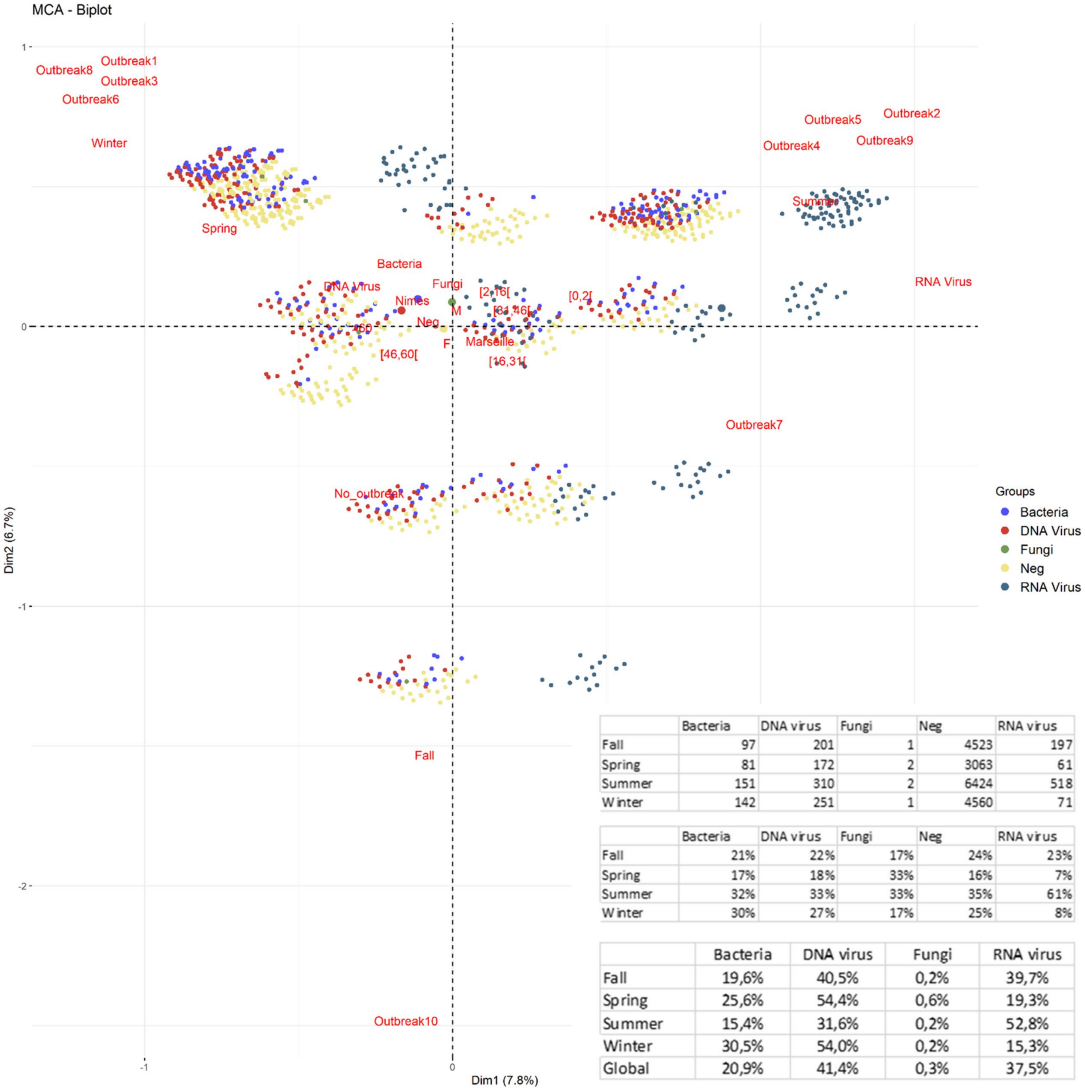
Multiple correspondence analysis (MCA) of the variables of seasons, outbreak number, identified pathogens gathered by microorganisms’ groups (RNA virus, DNA virus, Bacteria, and Fungi), gender, and age. In summer, five outbreaks caused by RNA viruses infecting young patients (61%), represented 52.8% (*p* < 10^−4^) of summer outbreaks. DNA viruses occurred in all seasons with a more equal distribution but accounted for ~54% (*p* < 10^−4^) of both spring and winter outbreaks. Although bacteria were less season-specific, they were nevertheless more frequent in winter (30% of bacteria were detected in winter) and accounted for 30.5% of winter epidemics (*p* < 10^−4^). According to the ACM, RNA viruses are the furthest from the center of the benchmark and are associated with summer. Bacteria and DNA viruses are closer to the center, so less season-specific, but still closer to winter and spring. Undocumented cases mainly clustered with bacteria and DNA viruses in winter and with all etiologies in summer, notably with RNA viruses, especially for young patients. *Fisher’s Exact Test for Count Data with simulated *p* value (based on 2,000 replicates), (*p* value = 0.0004998).

Each outbreak included 991–1,834 (median, 1,289) patients, of whom only 100–310 (median, 125) were documented; this ratio of 11.3% was significantly higher than the 10.1% ratio found in outside outbreaks (*p* = 0.01; Figure 4). Specifically, outbreak-1 (January to Mid-March 2015) included 100/1,292 (7.7%) documented patients infected by HSV-1 (29%), HSV-2 (8%), VZV (12%), *S. pneumoniae* (9%), *N. meningitidis* (5%), and Enterovirus (8%); outbreak-2 (April to July 2015) included 141/1,103 (12.8%) documented patients mainly infected by Enterovirus (64.5%), HSV-1 (10.6%), and VZV (9.9%), which affected children aged <16 years old in 45.4% of cases; outbreak-3 (January to mid-March 2016) included 102/1,212 (8.4%) documented patients mainly infected by HSV-1 (27.4%), Enterovirus (21.6%), VZV (14.7%), *S. pneumoniae* (8.8%), *N. meningitidis* (4.9%), and HHV-6 (4.9%); outbreak-4 (March to July 2016) included 118/1,321 (8.94%) documented patients, of which 44.1% of infections were caused by Enterovirus; outbreak-5 (March to July 2017) included 205/1,399 (14.6%) documented patients, of which 70% of infections were caused by Enterovirus; outbreak-6 (November 2017 to February 2018) included 132/1366 (9.7%) documented patients with infections caused by HSV-1 (27.3%), HSV-2 (10.6%), VZV (11.4%), *S. pneumoniae* (9.1%), Enterovirus (9.1%), HHV-6 (8.3%), *N. meningitidis* (5.3%), and *S. agalactiae* (3.8%); outbreak-7 (May to September 2018) included 310/1,834 (16.9%) documented patients with infections caused by Enterovirus (50.6%), and VZV (10%), HSV-1 (8.4%), and Human parechovirus (7.1%); outbreak-8 (November 2018 to February 2019) included 117/1,286 (9.11%) documented patients infected by HSV-1 (31.6%), VZV (8.5%), *S. pneumoniae* (12%), Enterovirus (9.4%), and *H. influenza* (7.7%); and outbreak-9 (May to July 2019) included 137/991 (13.8%) documented patients mainly infected by Enterovirus (42.3%). Finally, outbreak-10 (August to October 2019) was the least documented outbreak, with only 109/1,245 (8.7%) documented patients infected, and > 70% of these cases were caused by one of several neurotropic viruses (Figure 4). In summary, winter outbreaks 1, 3, 6 and 8 included infections by HSV-1 (28.8%), HSV-2 (7.3%), VZV (11.5%), *S. pneumoniae* (9.7%), HHV-6 (6.2%), *N. meningitidis* (3.9%), *S. agalactiae* (2.2%), and *Tropheryma whipplei* (2%), leaving 91.2% of cases undocumented, while summer outbreaks 2, 4, 5, 7, and 9 included infections by Enterovirus and Human parechovirus (specifically in 2018) and left 86.48% of cases undocumented (Figures 3, 4). Incorporating the incidence of documented vs. undocumented cases, patient age, outbreak period, and season as variables, MCA clustered the occurrence of summer undocumented cases with patients aged <31 years old and infection with Enterovirus and Human parechovirus, and MCA clustered winter undocumented cases with patients aged >45 years old and infection with *N. meningitidis*, *S. pneumoniae*, HSV, VZV, and Cytomegalovirus (Figure 3). Outside outbreak periods, we observed a persistence of infection with HSV-2, HHV-6, and some bacteria, such as *H. influenzae*, and with other infrequent bacteria without any detected association with sex (Appendix 2). After etiologies were grouped, reanalysis of the data indicated that RNA viruses occurred significantly more frequently in summer, with a rapid and strong increase (61%, *p* < 10^−4^) and with 52.8% of outbreaks occurring in summer. DNA viruses were identified significantly more frequently in the winter/spring season (54%, *p* < 10^−4^), while no specific relationship between bacterial infection and season was observed, despite the significant increase in the frequency of bacterial infection during winter outbreaks (30.5%, *p* < 10^−4^; Figure 5). However, for outbreaks 7, 8, and 9, which occurred at the same time as outbreaks of RNA viruses in summer and autumn 2018 and summer 2019, there was no clear association between patient age and sex. The same pattern of a ~1 week displacement moving in the same direction was observed by superimposing temperature data. The one-week delay in temperature displacement from Nîmes to Marseille was significantly correlated (*p* < 0.001) with the 1-week delay in CAM outbreak displacement, whereas no such significant shift was observed for the “humidity” and “wind” variables (Figure 4B).

## Discussion

Retrospective investigation of a large series of CAMs in southern France yielded a complex, dynamic epidemiological pattern combining seemingly sporadic cases and clustered cases, later forming a total of 10 outbreaks occurring over 61 months of investigation (Broberg et al., 2018; Sigfrid et al., 2019). The investigation cases from two university hospitals in cities separated by 120 km but located in the same region, in which CAM cases were investigated using the same laboratory protocols, allowing for the unprecedented observation of CAM outbreak temporal and spatial dynamics, which were characterized in this region by an eastward displacement at an average of ~9 km/day. The same displacement pattern was observed with temperature records and correlated with outbreaks consistently originating in Nîmes before moving to Marseille, and these data provided the basis for an analysis of the influence of atmospheric temperature and geography on the dynamics of CAM outbreaks. Temperature was found to be significantly associated with the displacement dynamics of CAM outbreaks (Depaquit et al., 2010; Beauté et al., 2018; Broberg et al., 2018).

Whether temperature was just a marker for certain changing biological conditions in populations, pathogens, and vectors, or whether it was a direct biological determinant remains uncertain in this study. One unanticipated observation was that eight CAM syndrome outbreaks varied in the proportion of documented and nondocumented cases, while two outbreaks overwhelming comprised undocumented cases (Figure 4). Documented Enterovirus cases were responsible for six seasonal outbreaks among the 10 outbreaks observed here and affected young patient populations, as previously described (Faustini et al., 2006; Broberg et al., 2018; Reusken et al., 2019; Tschumi et al., 2019; Hobday et al., 2020; Nkosi et al., 2021; Zhan et al., 2021). Accordingly, in 2018 (Broberg et al., 2018), the unusual persistence of mixed Enterovirus and Human parechovirus outbreaks infecting new-borns and children until the autumn correlated with a notable 2°C increase in autumn temperatures in October/November 2018 compared to seasonal norms (Cabrerizo et al., 2015). Most intriguing was the observation that DNA viruses also adopted an outbreak pattern, with HSV-1 CAM being observed in elderly male patients in January–March, probably due to the HSV-1 reactivation (Dudgeon, 1969; Taylor et al., 2002), which is potentially prompted by vitamin D deficiency related to low sun exposure in winter, especially in people >70 years old, as previously reported (Lin et al., 2019, 2020; Cristian Ilie and Stefanescu, 2020). Accordingly, significant correlations between pathogen, gender and age differentially observed in Nimes and Marseille may simply reflect differences in the population structure in these two different cities.

By superimposing the characteristics of undocumented patients with documented ones, MCA shed light on at least two seasonal patterns for such undocumented outbreaks, opening up avenues for future research. In summer, undocumented cases closely clustered with the incidence of RNA viruses, whereas in fall and winter, they clustered with that of DNA viruses and bacteria. This trend could clarify that the increase in hospital admissions in the summer may be due to infection with RNA viruses, and the high admission of older patients in the autumn/winter season is probably due to infection with DNA viruses and/or bacteria which are not routinely investigated at the POC laboratories (Vincent et al., 2020). With the exception of some skin flora contaminants following either lumbar puncture or CSF tube manipulation, such as *Cutibacterium acnes* and *Staphylococcus epidermidis*, not all causative pathogens were routinely targeted at POC laboratories in this study, including emerging genotypes and arthropod-borne viruses escaping routine detection (Global Burden, 2015; Lin et al., 2019; Morsli et al., 2022). We propose that Enteroviruses and arthropod-borne viruses are two groups of candidate pathogens to be further examined to account for remaining undocumented cases. Furthermore, future studies may associate this dynamic with genomic data, particularly Enterovirus genotypes (Broberg et al., 2018), to determine the genotypes circulating in our region which are mainly involved in the outbreaks.

## Conclusion

This retrospective study shed light on the significant correlation between temperature and the occurrence of CAM outbreaks in southern France, indicating the need to develop new laboratory tools to search for probable RNA viruses responsible for the majority of currently undocumented cases of CAMs in summer and autumn in this region. Real-time metagenomics based on pathogen genome detection performed directly from CSF could be part of this new strategy, with the aim of reducing the number of undocumented CAMs (Morsli et al., 2022).

## Data availability statement

The original contributions presented in the study are included in the article/Supplementary material; further inquiries can be directed to the corresponding author.

## Author contributions

MM: data collection, data cleaning, design of the study, data interpreting, and validation and writing of the manuscript. FS: data cleaning, statistical analysis, data interpretation, and validation and writing of the manuscript. QK, AB, RS, CD-R, and CZ: clinical data collection and data interpretation. J-PL and MD: design of the study, data interpretation, validation, funding, critically reviewing of the manuscript, coordination, and directing the work. All authors contributed to the article and approved the submitted version.

## Funding

This study was supported by the Fondation Méditerranée Infection, IHU Méditerranée Infection, Marseille, France. MM is a PhD student supported by the Fondation Méditerranée Infection. This work was supported by the French Government under the Investissements d’Avenir (Investments in the Future) program managed by the Agence Nationale de la Recherche (ANR, fr: National Agency for Research; reference: Méditerranée Infection 10-IAHU-03).

## Conflict of interest

The authors declare that the research was conducted in the absence of any commercial or financial relationships that could be construed as a potential conflict of interest.

## Publisher’s note

All claims expressed in this article are solely those of the authors and do not necessarily represent those of their affiliated organizations, or those of the publisher, the editors and the reviewers. Any product that may be evaluated in this article, or claim that may be made by its manufacturer, is not guaranteed or endorsed by the publisher.

## Footnotes

^1^ https://www.paca.ars.sante.fr/surveillance-epidemiologique-des-infections-virus

^2^ https://www.historique-meteo.net/france/
