# Supplementary material for: Dynamics of community-acquired meningitis syndrome outbreaks in southern France
Source: Front Microbiol. 2023 Jan 26;13:1102130. doi: 10.3389/fmicb.2022.1102130 (PMC9909019; doi:10.3389/fmicb.2022.1102130)
Supplement: Supplementary file 1 [file Data_Sheet_1.docx]

**Supplementary information**


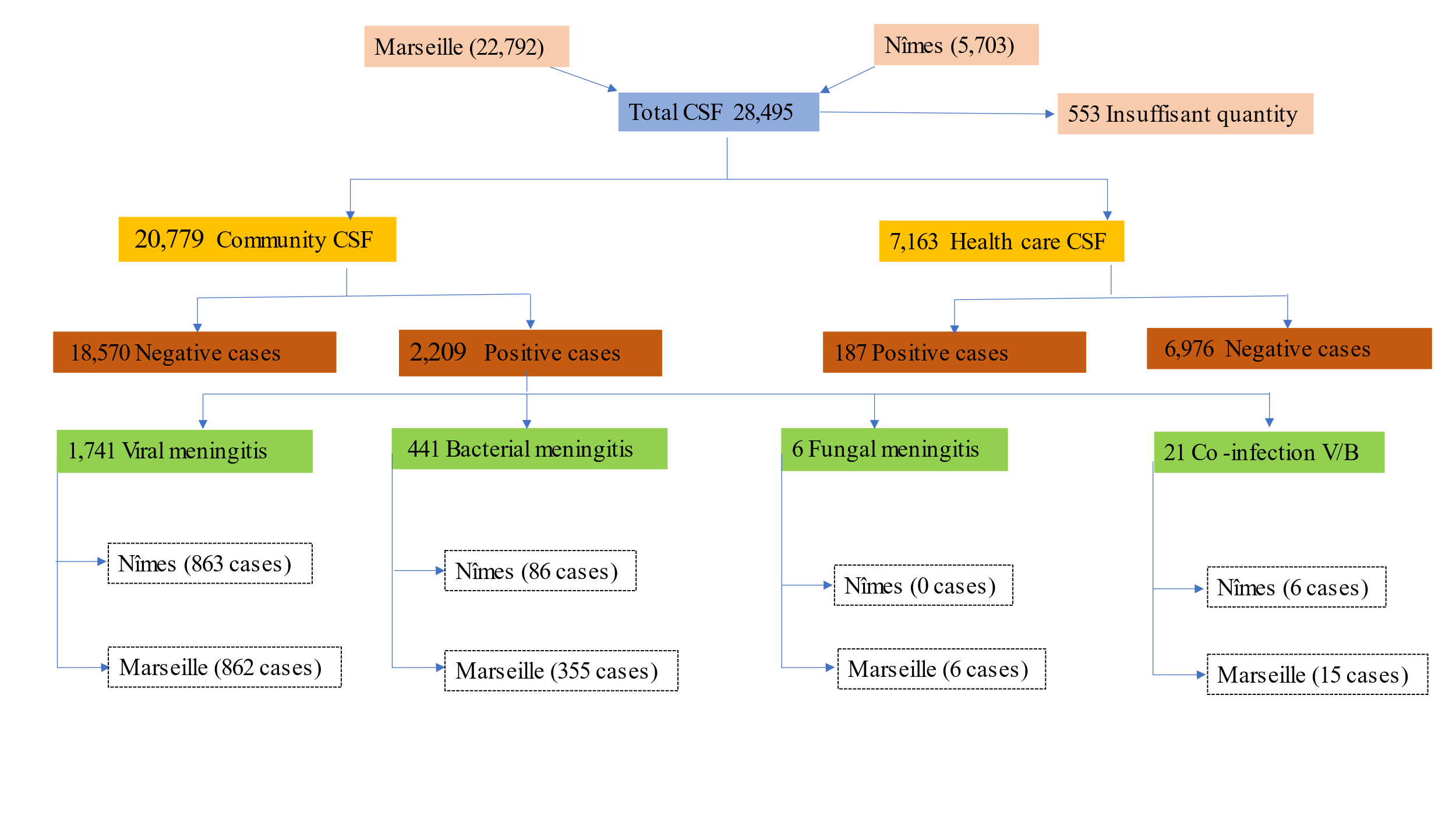


**Appendix 1**: Flow-chart of screening data included in the retrospective study of community-acquired meningitis syndrome collected in two university hospitals in southern France. All surgical cases, dialysis, transplantation, and hospitalization of more than 48 hours were excluded

**
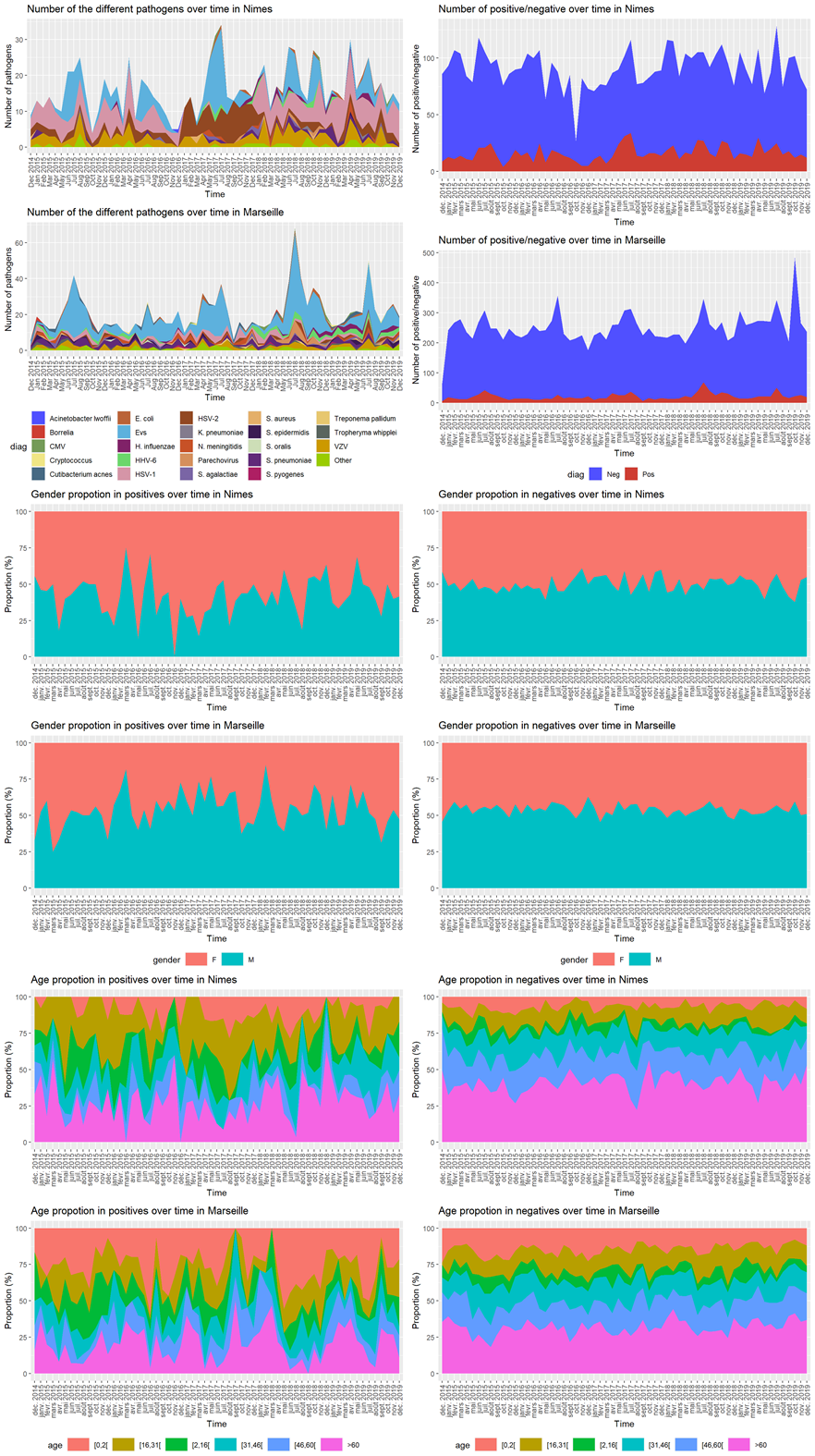
**

**Appendix 2:** Evolution over time (in proportion) of positivity (documented vs undocumented), pathogens, gender, and age classes. Left part graphics give characteristics of documented samples whereas right part gives characteristics of undocumented samples. Both Nimes (top) and Marseille (bottom) are represented for each situation.


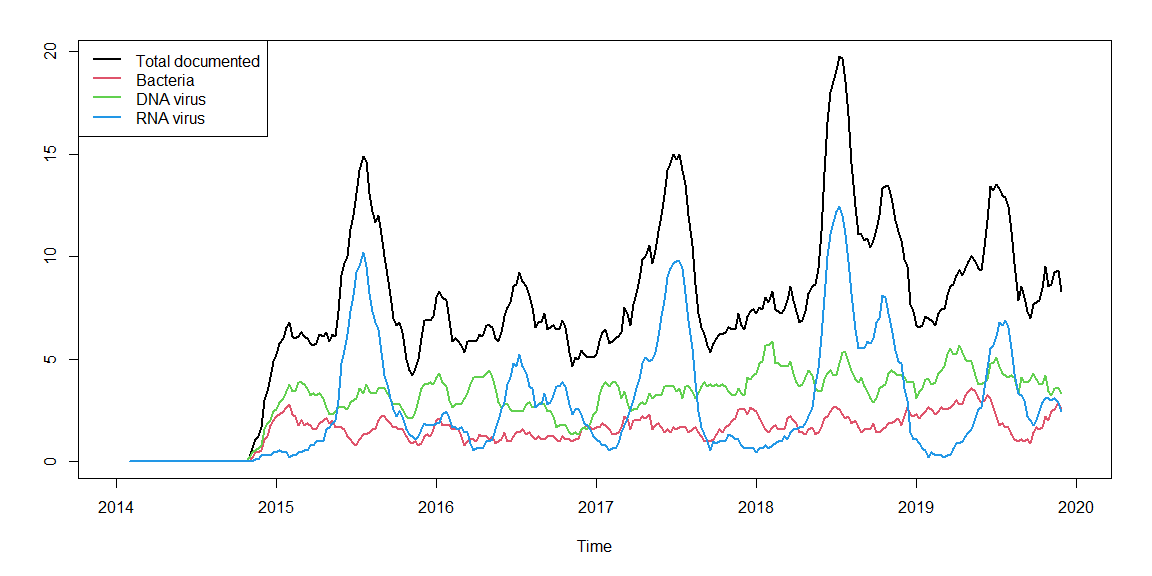


**Appendix 3**: Smoothed (moving average of size 9 weeks) time series of total documented, DNA viruses, RNA viruses and bacteria.
